# Supplementary figures and images for: Profiles of oral microbiota and metabolites in periodontitis and benign prostatic hyperplasia patients: a pilot study
Source: Microbiol Spectr. 2025 Aug 19;13(10):e03376-24. doi: 10.1128/spectrum.03376-24 (PMC12502708; doi:10.1128/spectrum.03376-24)

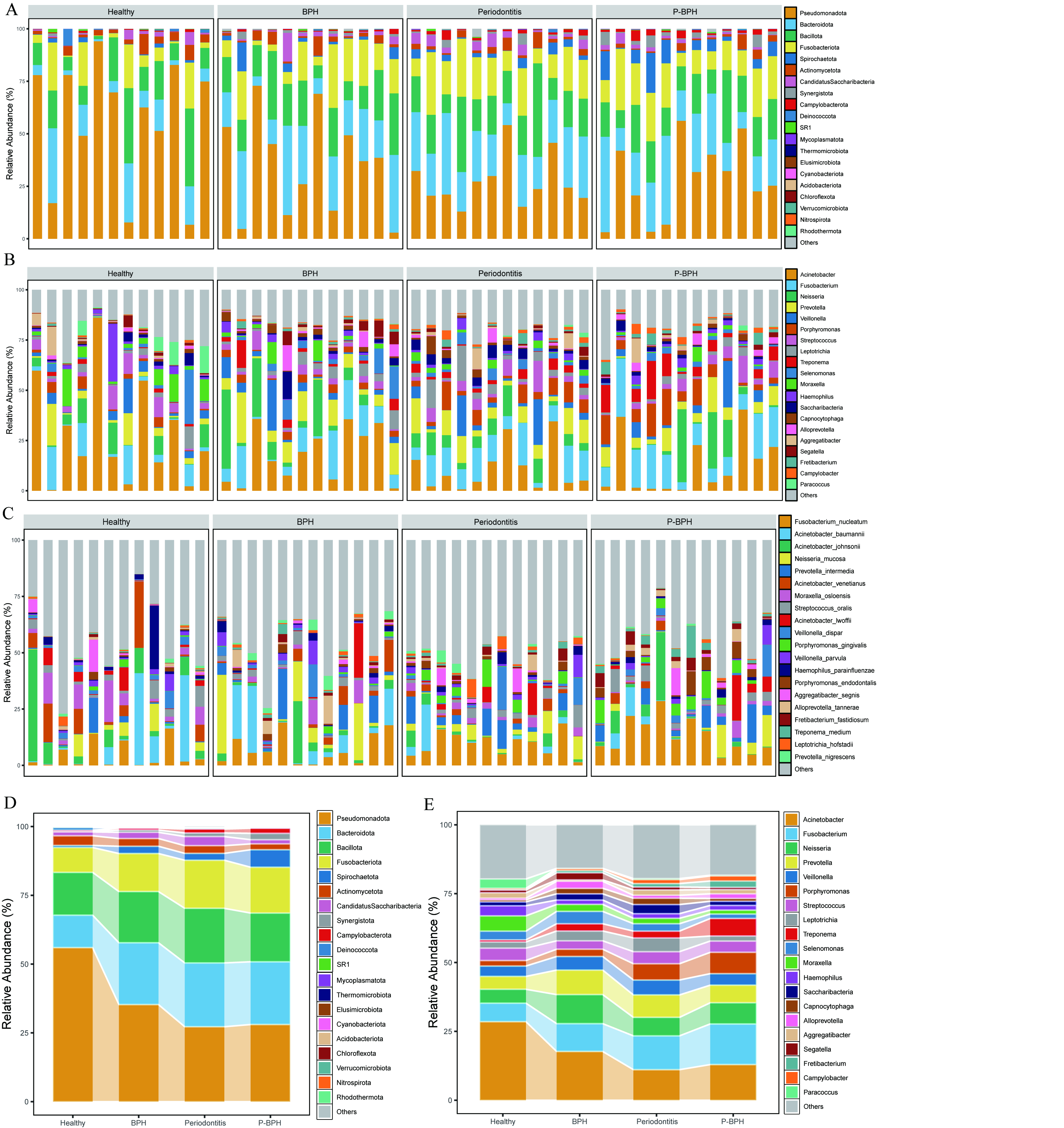

Supplement: Fig. S1 — Species composition of oral microbiota among the Healthy, BPH, Periodontitis, and P-BPH groups. [file spectrum.03376-24-s0001.tif]

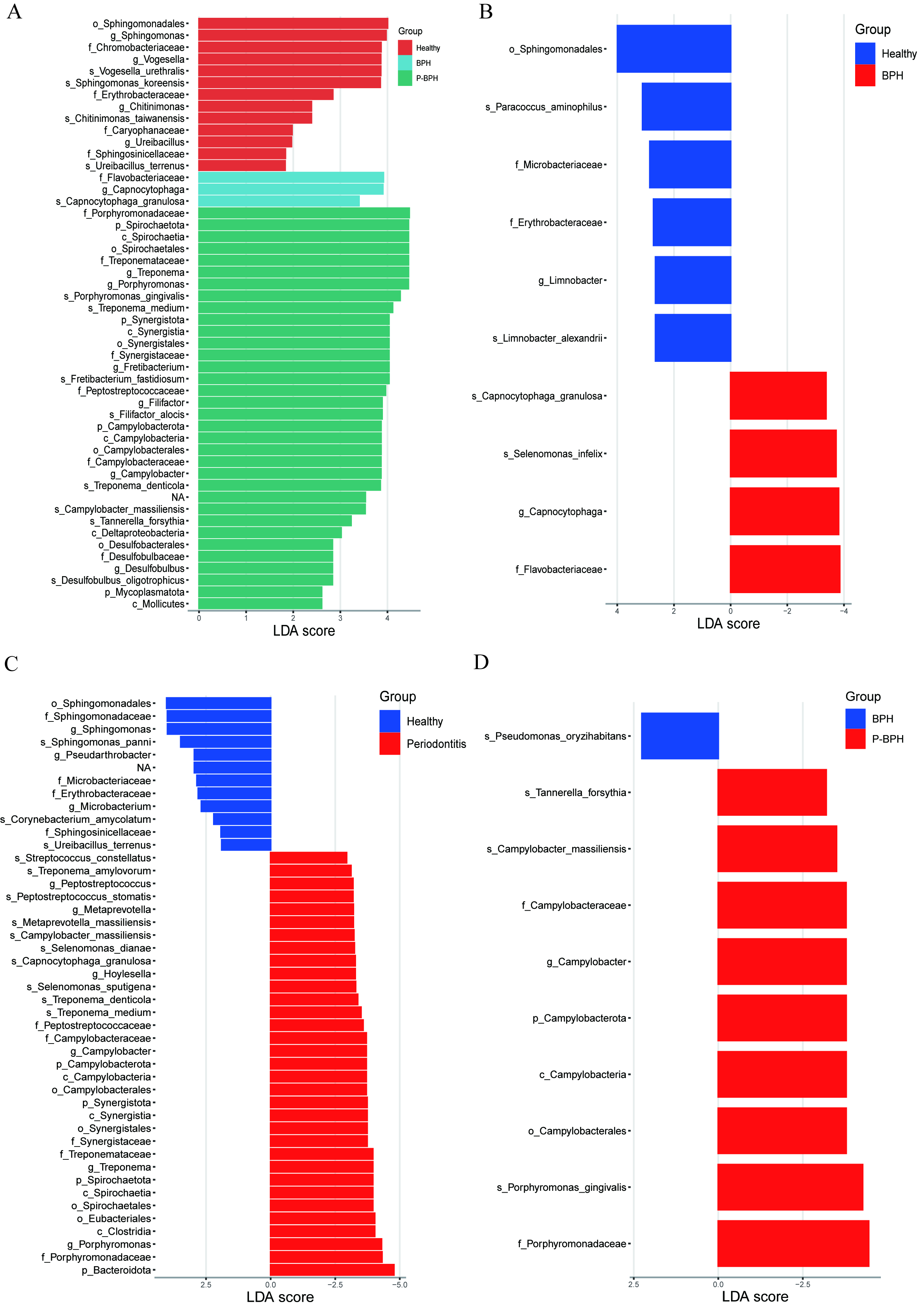

Supplement: Fig. S2 — LDA effect size (LEfSe) analysis was used to compare the different microorganisms among the four groups. [file spectrum.03376-24-s0002.tif]

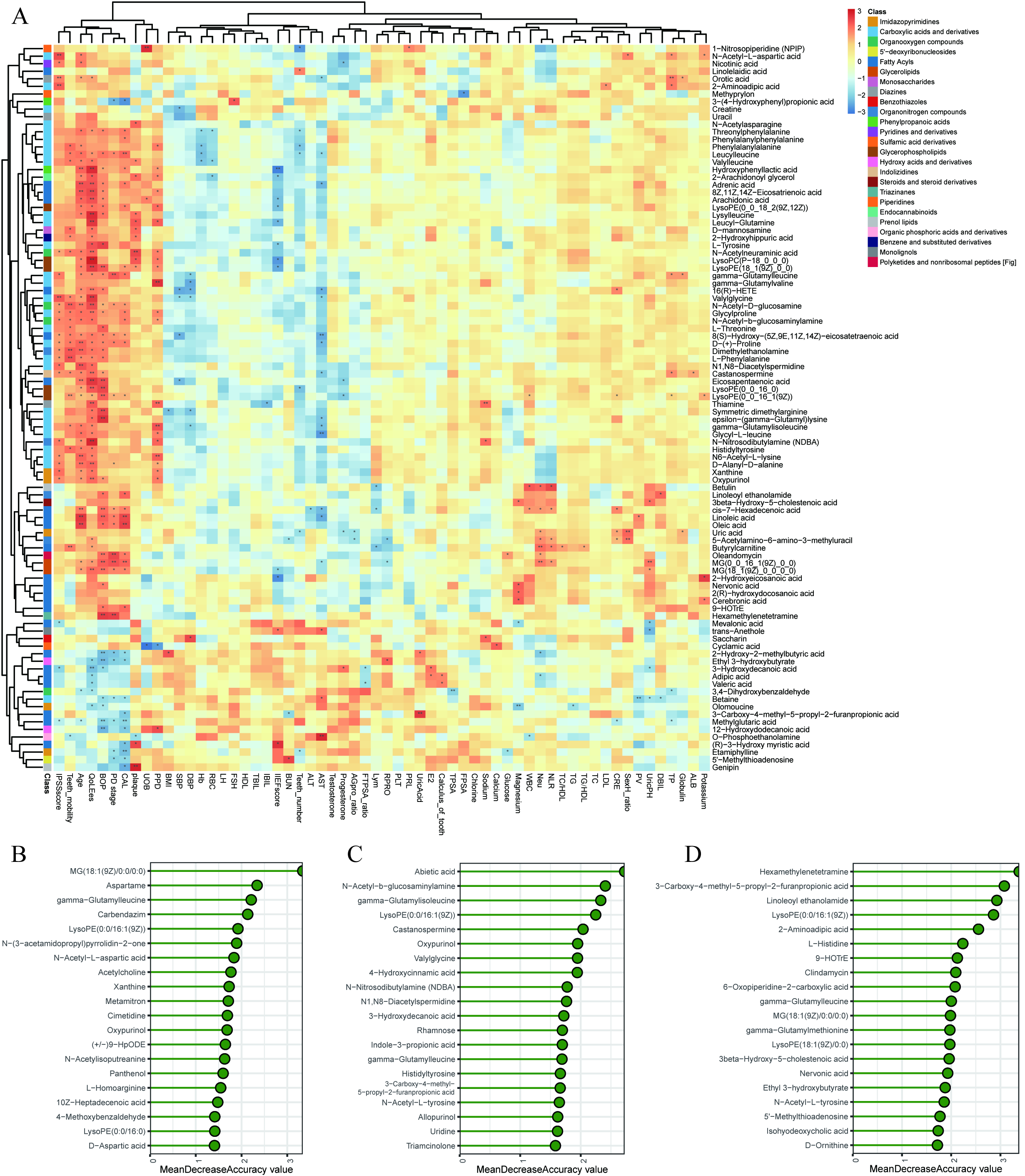

Supplement: Fig. S3 — Correlation between differential metabolite and clinical parameters. [file spectrum.03376-24-s0003.tif]
