# Supplementary material for: Profiles of oral microbiota and metabolites in periodontitis and benign prostatic hyperplasia patients: a pilot study
Source: Microbiol Spectr. 2025 Aug 19;13(10):e03376-24. doi: 10.1128/spectrum.03376-24 (PMC12502708; doi:10.1128/spectrum.03376-24)
Supplement: Supplemental material — Supplemental figure legends. [file spectrum.03376-24-s0004.docx]

Fig S1. Species composition of oral microbiota among the Healthy, BPH, Periodontitis, and P-BPH groups. A. Species distribution of each sample at the phylum level. B. Species distribution of each sample at the genus level. C. Species distribution of each sample at the species level. D. Species composition at the phylum level among the groups. E. Species composition at the genus level among the groups.

Fig S2. Linear discriminant analysis (LDA) effect size (LEfSe) analysis was used to compare the different microorganisms among the four groups, with an LDA cutoff >2. A. Comparison among the four groups; B. BPH group versus healthy group; C. Periodontitis group versus healthy group; D. P-BPH group versus BPH group. p_: Phylum, c_: Class, o_: Order, f_: family, g_: Genus, s_: Species.

Fig S3 Correlation between differential metabolite and clinical parameters A. Heat map of Spearman correlation coefficient between four groups of oral differential metabolite and clinical information. Random forest model was used to identify the most important microbial features in various comparisons: B. Periodontitis versus Healthy; C. BPH versus Healthy; C. P-BPH versus healthy. BMI: Body mass index, SexH_ratio: Estradiol to testosterone ratio, TG: Triglycerides, HDL: High density lipoprotein, TC: Total cholesterol, PV: Prostate volume, PPD: Probing pocket depth, UOB: Urine occult blood, E2: Estradiol, LH: Luteinizing hormone, AST: Aspartate transaminase, ALT: Alanine transaminase, BUN: Blood urea nitrogen, DBP: Diastolic blood pressure, SBP: Systolic blood pressure, FPSA: Free prostate-specific antigen, TPSA: Total prostate-specific antigen, RBC: Red blood cell count, NLR: Neutrophil to lymphocyte ratio, Neu: Neutrophil percentage, PLT: Platelet count, FSH: Follicle stimulating hormone, WBC: White blood cell count, UricPH: Urine pH, AGpro_ratio: Albumin/Globulin, LDL: Low density lipoprotein, PRL: Prolactin, ALB: Albumin, FTPSA_ratio: FPSA/TPSA, CRE: Creatinine, TP: Total protein, DBIL: Direct bilirubin, IIEF: International index of erectile function, RPRO: Urine protein, Lym: Lymphocyte percentage, IBIL: Indirect bilirubin, TBIL: Total bilirubin, QoL: Quality of life, IPSS: International prostate symptom score, CAL: Clinical attachment loss, PD stage: Periodontitis stage, BOP: Bleeding on probing.
