# Supplementary material for: Profiles of oral microbiota and metabolites in periodontitis and benign prostatic hyperplasia patients: a pilot study
Source: Microbiol Spectr. 2025 Aug 19;13(10):e03376-24. doi: 10.1128/spectrum.03376-24 (PMC12502708; doi:10.1128/spectrum.03376-24)
Supplement: Supplemental tables — Tables S1 to S3. [file spectrum.03376-24-s0005.docx]

**Table S1. Clinical parameters of PD and BPH groups relative to healthy and combined groups**

| **Parameters** | **Healthy** | **PD** | *P* value | **Healthy** | **BPH** | *P* value | **Healthy** | **P_BPH** | *P* value | **PD** | **P_BPH** | *P* value | **BPH** | **P_BPH** | *P* value |
| --- | --- | --- | --- | --- | --- | --- | --- | --- | --- | --- | --- | --- | --- | --- | --- |
| **(mean±SD; n%)** | **(n=12)** | **(n=12)** |  | **(n=12)** | **(n=12)** |  | **(n=12)** | **(n=12)** |  | **(n=12)** | **(n=12)** |  | **(n=12)** | **(n=12)** |  |
| **Age** | 46.25 (±2.83) | 47.33 (±4.05) | 0.456 | 46.25 (±2.83) | 46.75 (±4.14) | 0.733 | 46.25 (±2.83) | 50.08 (±4.03) | 0.013 | 47.33 (±4.05) | 50.08 (±4.03) | 0.11 | 46.75 (±4.14) | 50.08 (±4.03) | 0.058 |
| **BMI** | 24.63 (±3.68) | 25.66 (±3.35) | 0.483 | 24.63 (±3.68) | 24.94 (±3.56) | 0.837 | 24.63 (±3.68) | 24.66 (±2.85) | 0.985 | 25.66 (±3.35) | 24.66 (±2.85) | 0.44 | 24.94 (±3.56) | 24.66 (±2.85) | 0.832 |
| **Education** | | | | | | | | | | | | | | | |
| College or above | 6 (50.0) | 6 (50.0) | 1 | 6 (50.0) | 6 (50.0) | 1 | 6 (50.0) | 3 (25.0) | 0.399 | 6 (50.0) | 3 (25.0) | 0.399 | 6 (50.0) | 3 (25.0) | 0.399 |
| High school or below | 6 (50.0) | 6 (50.0) |  | 6 (50.0) | 6 (50.0) |  | 6 (50.0) | 9 (75.0) |  | 6 (50.0) | 9 (75.0) |  | 6 (50.0) | 9 (75.0) |  |
| **Smoking** | | | | | | | | | | | | | | | |
| smoker | 5 (41.7) | 3 (25.0) | 0.665 | 5 (41.7) | 2 (16.7) | 0.369 | 5 (41.7) | 4 (33.3) | 1 | 3 (25.0) | 4 (33.3) | 1 | 2 (16.7) | 4 (33.3) | 0.637 |
| Non-smoker | 7 (58.3) | 9 (75.0) |  | 7 (58.3) | 10 (83.3) |  | 7 (58.3) | 8 (66.7) |  | 9 (75.0) | 8 (66.7) |  | 10 (83.3) | 8 (66.7) |  |
| **Drinking** | | | | | | | | | | | | | | | |
| Non-drinker | 6 (50.0) | 3 (25.0) | 0.399 | 6 (50.0) | 2 (16.7) | 0.194 | 6 (50.0) | 6 (50.0) | 1 | 3 (25.0) | 6 (50.0) | 0.399 | 2 (16.7) | 6 (50.0) | 0.194 |
| drinker | 6 (50.0) | 9 (75.0) |  | 6 (50.0) | 10 (83.3) |  | 6 (50.0) | 6 (50.0) |  | 9 (75.0) | 6 (50.0) |  | 10 (83.3) | 6 (50.0) |  |
| **Tea** | | | | | | | | | | | | | | | |
| Non-tea drinker | 5 (41.7) | 1 (8.3) | 0.157 | 5 (41.7) | 2 (16.7) | 0.369 | 5 (41.7) | 2 (16.7) | 0.369 | 1 (8.3) | 2 (16.7) | 1 | 2 (16.7) | 2 (16.7) | 1 |
| Tea Drinker | 7 (58.3) | 11 (91.7) |  | 7 (58.3) | 10 (83.3) |  | 7 (58.3) | 10 (83.3) |  | 11 (91.7) | 10 (83.3) |  | 10 (83.3) | 10 (83.3) |  |
| **PV (mL)** | 17.47 (±3.31) | 19.04 (±5.93) | 0.43 | 17.47 (±3.31) | 24.34 (±9.65) | 0.03 | 17.47 (±3.31) | 28.52 (±11.64) | 0.005 | 19.04 (±5.93) | 28.52 (±11.64) | 0.021 | 24.34 (±9.65) | 28.52 (±11.64) | 0.37 |
| **IPSS** | 2.58 (±2.02) | 3.25 (±2.30) | 0.459 | 2.58 (±2.02) | 9.67 (±5.93) | 0.001 | 2.58 (±2.02) | 7.83 (±9.11) | 0.064 | 3.25 (±2.30) | 7.83 (±9.11) | 0.105 | 9.67 (±5.93) | 7.83 (±9.11) | 0.565 |
| **IIEF** | 19.25 (±2.96) | 19.42 (±3.26) | 0.897 | 19.25 (±2.96) | 19.00 (±3.44) | 0.85 | 19.25 (±2.96) | 18.82 (±3.89) | 0.766 | 19.42 (±3.26) | 18.82 (±3.89) | 0.693 | 19.00 (±3.44) | 18.82 (±3.89) | 0.906 |
| **QOL** | 1.42 (±1.44) | 1.92 (±0.90) | 0.32 | 1.42 (±1.44) | 2.83 (±1.27) | 0.018 | 1.42 (±1.44) | 3.08 (±1.44) | 0.01 | 1.92 (±0.90) | 3.08 (±1.44) | 0.027 | 2.83 (±1.27) | 3.08 (±1.44) | 0.656 |
| **PSA (ng/mL)** | 0.88 (±0.37) | 0.93 (±0.57) | 0.793 | 0.88 (±0.37) | 1.26 (±0.82) | 0.158 | 0.88 (±0.37) | 1.12 (±0.73) | 0.319 | 0.93 (±0.57) | 1.12 (±0.73) | 0.485 | 1.26 (±0.82) | 1.12 (±0.73) | 0.672 |
| **FPSA (ng/mL)** | 0.23 (±0.09) | 0.25 (±0.20) | 0.71 | 0.23 (±0.09) | 0.30 (±0.18) | 0.207 | 0.23 (±0.09) | 0.30 (±0.17) | 0.212 | 0.25 (±0.20) | 0.30 (±0.17) | 0.528 | 0.30 (±0.18) | 0.30 (±0.17) | 0.965 |
| **FPSA/PSA** | 0.26 (±0.10) | 0.27 (±0.11) | 0.969 | 0.26 (±0.10) | 0.28 (±0.12) | 0.742 | 0.26 (±0.10) | 0.30 (±0.12) | 0.46 | 0.27 (±0.11) | 0.30 (±0.12) | 0.505 | 0.28 (±0.12) | 0.30 (±0.12) | 0.705 |
| **Teeth number** | 29.00 (±1.60) | 28.75 (±1.71) | 0.715 | 29.00 (±1.60) | 28.75 (±1.86) | 0.728 | 29.00 (±1.60) | 28.58 (±2.11) | 0.591 | 28.75 (±1.71) | 28.58 (±2.11) | 0.834 | 28.75 (±1.86) | 28.58 (±2.11) | 0.839 |
| **BOP** | 0.12 (±0.17) | 0.35 (±0.26) | 0.019 | 0.12 (±0.17) | 0.09 (±0.10) | 0.636 | 0.12 (±0.17) | 0.53 (±0.29) | <0.001 | 0.35 (±0.26) | 0.53 (±0.29) | 0.118 | 0.09 (±0.10) | 0.53 (±0.29) | <0.001 |
| **CAL (mm)** | 0.02 (±0.05) | 1.25 (±0.98) | <0.001 | 0.02 (±0.05) | 0.03 (±0.07) | 0.63 | 0.02 (±0.05) | 1.22 (±0.94) | <0.001 | 1.25 (±0.98) | 1.22 (±0.94) | 0.936 | 0.03 (±0.07) | 1.22 (±0.94) | <0.001 |
| **PPD (mm)** | 2.34 (±0.37) | 2.27 (±0.47) | 0.678 | 2.34 (±0.37) | 2.56 (±0.48) | 0.22 | 2.34 (±0.37) | 2.63 (±0.62) | 0.186 | 2.27 (±0.47) | 2.63 (±0.62) | 0.126 | 2.56 (±0.48) | 2.63 (±0.62) | 0.783 |
| **PD stage** | 0.00 (0.00) | 2.42 (0.79) | <0.001 | 0.00 (0.00) | 0.00 (0.00) | NA | 0.00 (0.00) | 2.00 (0.85) | <0.001 | 2.42 (0.79) | 2.00 (0.85) | 0.228 | 0.00 (0.00) | 2.00 (0.85) | <0.001 |

BMI: Body mass index, PV: Prostate volume, IPSS: International prostate symptom score, IIEF: International index of erectile function, QoL: Quality of life, PSA: Prostate specific antigen, FPSA: Free prostate specific antigen, BOP: Bleeding on probing, CAL: Clinical attachment level, PPD: Probing pocket depth, PD stage: Periodontitis stage.

**Table S2. Differential oral microbiome among Periodontitis, BPH, P-BPH, and Healthy Groups.**

| **Group** | **Differential bacteria** | **log2FC** | **genus** | **P value** |
| --- | --- | --- | --- | --- |
| **BPH versus Healthy** | *Selenomonas sputigena* | 2.73 | *Selenomonas* | 0.04 |
|  | *Capnocytophaga granulosa* | 2.22 | *Capnocytophaga* | 0.00 |
|  | *Selenomonas infelix* | 2.13 | *Selenomonas* | 0.01 |
|  | *Neisseria elongata* | 2.03 | *Neisseria* | 0.03 |
|  | *Capnocytophaga leadbetteri* | 1.77 | *Capnocytophaga* | 0.03 |
|  | *Capnocytophaga ochracea* | 1.61 | *Capnocytophaga* | 0.01 |
|  | *Eikenella halliae* | 1.33 | *Eikenella* | 0.02 |
|  | *Treponema maltophilum* | 0.82 | *Treponema* | 0.03 |
|  | *Moraxella osloensis* | -1.29 | *Moraxella* | 0.04 |
|  | *Brevibacterium pityocampae* | -1.50 | *Brevibacterium* | 0.04 |
|  | *Kytococcus sedentarius* | -1.81 | *Kytococcus* | 0.04 |
|  | *Brachybacterium faecium* | -2.40 | *Brachybacterium* | 0.02 |
|  | *Paracoccus aminophilus* | -2.88 | *Paracoccus* | 0.01 |
|  | *Brevundimonas subvibrioides* | -5.77 | *Brevundimonas* | 0.05 |
|  | *Sphingomonas koreensis* | -5.99 | *Sphingomonas* | 0.04 |
|  | *Roseateles saccharophilus* | -6.40 | *Roseateles* | 0.02 |
|  | *Chitinimonas taiwanensis* | -8.07 | *Chitinimonas* | 0.03 |
|  | *Limnobacter alexandrii* | -29.82 | *Limnobacter* | 0.01 |
| **Periodontitis versus Healthy** | *Bacteroides heparinolyticus* | 3.62 | *Bacteroides* | 0.02 |
|  | *Metaprevotella massiliensis* | 3.43 | *Metaprevotella* | 0.01 |
|  | *Treponema amylovorum* | 3.30 | *Treponema* | 0.01 |
|  | *Fretibacterium fastidiosum* | 2.66 | *Fretibacterium* | 0.01 |
|  | *Peptoanaerobacter stomatis* | 2.52 | *Peptoanaerobacter* | 0.03 |
|  | *Streptococcus constellatus* | 2.37 | *Streptococcus* | 0.00 |
|  | *Porphyromonas gingivalis* | 2.37 | *Porphyromonas* | 0.02 |
|  | *Campylobacter gracilis* | 2.22 | *Campylobacter* | 0.04 |
|  | *Eikenella halliae* | 2.13 | *Eikenella* | 0.01 |
|  | *Treponema medium* | 2.08 | *Treponema* | 0.00 |
|  | *Treponema denticola* | 2.06 | *Treponema* | 0.01 |
|  | *Selenomonas dianae* | 1.99 | *Selenomonas* | 0.00 |
|  | *Capnocytophaga granulosa* | 1.94 | *Capnocytophaga* | 0.00 |
|  | *Filifactor alocis* | 1.79 | *Filifactor* | 0.01 |
|  | *Leptotrichia hofstadii* | 1.65 | *Leptotrichia* | 0.02 |
|  | *Stomatobaculum longum* | 1.65 | *Stomatobaculum* | 0.02 |
|  | *Peptostreptococcus stomatis* | 1.52 | *Peptostreptococcus* | 0.00 |
|  | *Campylobacter massiliensis* | 1.52 | *Campylobacter* | 0.00 |
|  | *Johnsonella ignava* | 1.45 | *Johnsonella* | 0.02 |
|  | *Selenomonas sputigena* | 1.37 | *Selenomonas* | 0.01 |
|  | *Selenomonas infelix* | 1.33 | *Selenomonas* | 0.01 |
|  | *Campylobacter concisus* | 1.24 | *Campylobacter* | 0.04 |
|  | *Leptotrichia buccalis* | 1.17 | *Leptotrichia* | 0.02 |
|  | *Schwartzia succinivorans* | 1.14 | *Schwartzia* | 0.03 |
|  | *Porphyromonas catoniae* | 1.09 | *Porphyromonas* | 0.03 |
|  | *Fusobacterium nucleatum* | 1.02 | *Fusobacterium* | 0.03 |
|  | *Treponema socranskii* | 0.74 | *Treponema* | 0.02 |
|  | *Brachybacterium faecium* | -1.91 | *Brachybacterium* | 0.02 |
|  | *Kytococcus sedentarius* | -1.93 | *Kytococcus* | 0.04 |
|  | *Paracoccus aminophilus* | -2.44 | *Paracoccus* | 0.03 |
|  | *Sphingomonas koreensis* | -3.86 | *Sphingomonas* | 0.02 |
|  | *Sphingomonas panni* | -4.77 | *Sphingomonas* | 0.00 |
| **P-BPH versus Healthy** | *Metaprevotella massiliensis* | 4.10 | *Metaprevotella* | 0.03 |
|  | *Treponema medium* | 3.89 | *Treponema* | 0.00 |
|  | *Treponema amylovorum* | 3.70 | *Treponema* | 0.00 |
|  | *Filifactor alocis* | 3.59 | *Filifactor* | 0.00 |
|  | *Fretibacterium fastidiosum* | 3.52 | *Fretibacterium* | 0.00 |
|  | *Treponema denticola* | 3.47 | *Treponema* | 0.00 |
|  | *Desulfobulbus oligotrophicus* | 3.34 | *Desulfobulbus* | 0.00 |
|  | *Tannerella forsythia* | 3.08 | *Tannerella* | 0.00 |
|  | *Porphyromonas gingivalis* | 2.81 | *Porphyromonas* | 0.01 |
|  | *Campylobacter gracilis* | 2.43 | *Campylobacter* | 0.04 |
|  | *Campylobacter massiliensis* | 2.38 | *Campylobacter* | 0.00 |
|  | *Treponema maltophilum* | 2.26 | *Treponema* | 0.00 |
|  | *Porphyromonas endodontalis* | 2.21 | *Porphyromonas* | 0.02 |
|  | *Dialister pneumosintes* | 2.19 | *Dialister* | 0.02 |
|  | *Capnocytophaga granulosa* | 1.89 | *Capnocytophaga* | 0.01 |
|  | *Johnsonella ignava* | 1.88 | *Johnsonella* | 0.01 |
|  | *Peptococcus simiae* | 1.83 | *Peptococcus* | 0.01 |
|  | *Fusobacterium nucleatum* | 1.46 | *Fusobacterium* | 0.00 |
|  | *Peptostreptococcus stomatis* | 1.35 | *Peptostreptococcus* | 0.04 |
|  | *Prevotella intermedia* | 1.32 | *Prevotella* | 0.03 |
|  | *Treponema socranskii* | 1.15 | *Treponema* | 0.02 |
|  | *Selenomonas sputigena* | 1.13 | *Selenomonas* | 0.04 |
|  | *Catonella morbi* | 0.69 | *Catonella* | 0.04 |
|  | *Bacteroides uniformis* | -1.16 | *Bacteroides* | 0.03 |
|  | *Faecalibacterium longum* | -1.20 | *Faecalibacterium* | 0.05 |
|  | *Kytococcus sedentarius* | -1.21 | *Kytococcus* | 0.03 |
|  | *Paracoccus aminophilus* | -1.50 | *Paracoccus* | 0.02 |
|  | *Moraxella osloensis* | -2.00 | *Moraxella* | 0.02 |
|  | *Bacteroides stercoris* | -2.52 | *Bacteroides* | 0.01 |
|  | *Acinetobacter venetianus* | -2.58 | *Acinetobacter* | 0.02 |
|  | *Acinetobacter variabilis* | -2.76 | *Acinetobacter* | 0.04 |
|  | *Pseudomonas aeruginosa* | -5.02 | *Pseudomonas* | 0.01 |
|  | *Sphingomonas koreensis* | -5.56 | *Sphingomonas* | 0.00 |
|  | *Deinococcus geothermalis* | -6.20 | *Deinococcus* | 0.03 |
|  | *Comamonas aquatica* | -6.55 | *Comamonas* | 0.02 |
|  | *Roseateles saccharophilus* | -6.99 | *Roseateles* | 0.00 |
|  | *Pseudomonas oryzihabitans* | -8.49 | *Pseudomonas* | 0.05 |
|  | *Vogesella urethralis* | -9.77 | *Vogesella* | 0.00 |
|  | *Chitinimonas taiwanensis* | -28.84 | *Chitinimonas* | 0.01 |
| **P-BPH versus BPH** | *Porphyromonas gingivalis* | 2.76 | *Porphyromonas* | 0.01 |
|  | *Tannerella forsythia* | 2.18 | *Tannerella* | 0.00 |
|  | *Campylobacter massiliensis* | 1.80 | *Campylobacter* | 0.00 |
|  | *Treponema medium* | 1.77 | *Treponema* | 0.03 |
|  | *Fretibacterium fastidiosum* | 1.52 | *Fretibacterium* | 0.02 |
|  | *Filifactor alocis* | 1.37 | *Filifactor* | 0.04 |
|  | *Desulfobulbus oligotrophicus* | 1.03 | *Desulfobulbus* | 0.04 |
|  | *Capnocytophaga ochracea* | -1.03 | *Capnocytophaga* | 0.03 |
|  | *Leptotrichia hongkongensis* | -1.48 | *Leptotrichia* | 0.01 |
|  | *Pseudomonas aeruginosa* | -1.84 | *Pseudomonas* | 0.01 |
|  | *Bacteroides stercoris* | -3.02 | *Bacteroides* | 0.04 |
|  | *Vogesella urethralis* | -3.50 | *Vogesella* | 0.03 |
|  | *Pseudomonas oryzihabitans* | -7.79 | *Pseudomonas* | 0.01 |
| **P-BPH versus PD** | *Filifactor alocis* | 1.80 | *Filifactor* | 0.02 |
|  | *Tannerella forsythia* | 1.23 | *Tannerella* | 0.01 |
|  | *Arachnia rubra* | -0.82 | *Arachnia* | 0.04 |
|  | *Lachnoanaerobaculum umeaense* | -1.03 | *Lachnoanaerobaculum* | 0.02 |
|  | *Campylobacter concisus* | -1.17 | *Campylobacter* | 0.02 |
|  | *Faecalibacterium longum* | -1.24 | *Faecalibacterium* | 0.01 |
|  | *Oribacterium asaccharolyticum* | -1.26 | *Oribacterium* | 0.02 |
|  | *Corynebacterium matruchotii* | -1.28 | *Corynebacterium* | 0.03 |
|  | *Enterobacter hormaechei* | -1.34 | *Enterobacter* | 0.03 |
|  | *Lancefieldella rimae* | -1.35 | *Lancefieldella* | 0.04 |
|  | *Hoylesella loescheii* | -1.35 | *Hoylesella* | 0.01 |
|  | *Phocaeicola dorei* | -1.50 | *Phocaeicola* | 0.01 |
|  | *Bacteroides uniformis* | -1.55 | *Bacteroides* | 0.03 |
|  | *Selenomonas noxia* | -1.70 | *Selenomonas* | 0.01 |
|  | *Leptotrichia hongkongensis* | -1.73 | *Leptotrichia* | 0.01 |
|  | *Roseburia inulinivorans* | -1.74 | *Roseburia* | 0.01 |
|  | *Porphyromonas catoniae* | -1.79 | *Porphyromonas* | 0.02 |
|  | *Tannerella serpentiformis* | -1.84 | *Tannerella* | 0.03 |
|  | *Leptotrichia hofstadii* | -1.91 | *Leptotrichia* | 0.04 |
|  | *Capnocytophaga sputigena* | -2.08 | *Capnocytophaga* | 0.00 |
|  | *Hoylesella nanceiensis* | -2.60 | *Hoylesella* | 0.02 |
|  | *Bacteroides stercoris* | -3.14 | *Bacteroides* | 0.02 |
|  | *Prevotella aurantiaca* | -3.83 | *Prevotella* | 0.04 |
|  | *Vogesella urethralis* | -6.67 | *Vogesella* | 0.03 |
|  | *Pseudomonas oryzihabitans* | -6.70 | *Pseudomonas* | 0.05 |

**Table S3 Significant differential metabolites among health, periodontitis, BPH, and P-BPH groups.**

| **Group** | **Class** | **Differential metabolites** | **log2FC** | **p value** |
| --- | --- | --- | --- | --- |
| BPH versus Healthy | Amines | Dimethylethanolamine | 0.88 | 0.05 |
|  | Amines and derivatives | LysoPE(0:0/16:0) | 2.15 | 0.01 |
|  |  | LysoPE(0:0/16:1(9Z)) | 1.87 | 0.04 |
|  | Amino acids, peptides, and analogues | Valylleucine | 2.27 | 0.04 |
|  |  | Lysylleucine | 2.10 | 0.04 |
|  |  | Histidyltyrosine | 1.97 | 0.01 |
|  |  | Leucylleucine | 1.79 | 0.03 |
|  |  | D-Alanyl-D-alanine | 1.57 | 0.01 |
|  |  | N-Acetyl-L-aspartic acid | 1.54 | 0.01 |
|  |  | Phenylalanylalanine | 1.36 | 0.02 |
|  |  | gamma-Glutamylleucine | 1.30 | 0.01 |
|  |  | Glycylproline | 1.11 | 0.01 |
|  |  | N6-Acetyl-L-lysine | 0.98 | 0.03 |
|  |  | Leucyl-Glutamine | 0.91 | 0.01 |
|  |  | N-Acetylasparagine | 0.90 | 0.01 |
|  |  | Valylglycine | 0.87 | 0.02 |
|  |  | Symmetric dimethylarginine | 0.77 | 0.05 |
|  | Benzene and derivatives | 2-Hydroxyhippuric acid | 1.06 | 0.04 |
|  | Benzothiazoles | Saccharin | -1.71 | 0.03 |
|  | Carbohydrates | N-Acetyl-b-glucosaminylamine | 1.43 | 0.01 |
|  |  | D-mannosamine | 1.41 | 0.02 |
|  | Fatty acyls[FA] | Adrenic acid | 3.85 | 0.03 |
|  |  | 8Z,11Z,14Z-Eicosatrienoic acid | 0.62 | 0.01 |
|  |  | Mevalonic acid | -2.49 | 0.05 |
|  | Glycerophospholipids[GP] | LysoPC(P-18:0/0:0) | 3.11 | 0.01 |
|  | Indolizidines | Castanospermine | 1.64 | 0.02 |
|  | Nitrosopiperidines | 1-Nitrosopiperidine (NPIP) | 0.72 | 0.04 |
|  | Organic acids | N1,N8-Diacetylspermidine | 2.07 | 0.01 |
|  |  | Cyclamic acid | -1.40 | 0.02 |
|  | Organic nitroso compounds | N-Nitrosodibutylamine (NDBA) | 0.93 | 0.02 |
|  | Phenylpropanoids | trans-Anethole | -1.06 | 0.03 |
|  | Purines and derivatives | Xanthine | 1.63 | 0.02 |
|  |  | Oxypurinol | 1.49 | 0.01 |
|  | Pyridine and derivatives | Nicotinic acid | 1.25 | 0.01 |
|  | Pyrimidines and pyrimidine derivatives | Thiamine | 2.63 | 0.02 |
|  |  | Orotic acid | 1.02 | 0.04 |
| Periodontitis versus Healthy | Amines and derivatives | LysoPE(0:0/16:1(9Z)) | 2.31 | 0.04 |
|  | Amino acids, peptides, and analogues | Leucylleucine | 2.23 | 0.02 |
|  |  | Histidyltyrosine | 2.12 | 0.03 |
|  |  | D-Alanyl-D-alanine | 1.22 | 0.02 |
|  |  | gamma-Glutamylleucine | 1.18 | 0.00 |
|  |  | gamma-Glutamylisoleucine | 1.10 | 0.02 |
|  | Benzene and derivatives | 2-Hydroxyhippuric acid | 1.19 | 0.03 |
|  | Carbohydrates | D-mannosamine | 1.96 | 0.01 |
|  |  | N-Acetyl-b-glucosaminylamine | 0.70 | 0.03 |
|  | Fatty acyls[FA] | Linolelaidic acid | 1.05 | 0.04 |
|  |  | cis-7-Hexadecenoic acid | 0.97 | 0.03 |
|  |  | Linoleic acid | 0.85 | 0.05 |
|  | Glycerolipids[GL] | MG(0:0/16:1(9Z)/0:0) | 0.97 | 0.03 |
|  | Organic acids | N1,N8-Diacetylspermidine | 1.56 | 0.03 |
|  | Phenylpropanoic acids | 3-(4-Hydroxyphenyl)propionic acid | -0.73 | 0.02 |
| P-BPH versus BPH | Antibiotics | Oleandomycin | 2.77 | 0.00 |
|  | Fatty acyls[FA] | 2-Hydroxy-2-methylbutyric acid | -1.13 | 0.03 |
|  | Glycerolipids[GL] | MG(0:0/16:1(9Z)/0:0) | 1.16 | 0.00 |
|  | Organic acids | Cyclamic acid | 1.09 | 0.02 |
|  |  | Ethyl 3-hydroxybutyrate | -0.60 | 0.01 |
|  | Piperidinones | Methyprylon | 1.10 | 0.02 |
|  | Purines and derivatives | Uric acid | 1.26 | 0.01 |
| P-BPH versus Periodontitis | Amines and derivatives | LysoPE(18:1(9Z)/0:0) | 0.90 | 0.04 |
|  | Amino acids | L-Tyrosine | 1.28 | 0.02 |
|  | Amino acids, peptides, and analogues | epsilon-(gamma-Glutamyl)lysine | 1.73 | 0.03 |
|  |  | Creatine | 0.62 | 0.04 |
|  | Fatty acyls[FA] | Nervonic acid | 1.93 | 0.02 |
|  |  | Eicosapentaenoic acid | 0.81 | 0.02 |
|  | N-arylamides | 5-Acetylamino-6-amino-3-methyluracil | 1.99 | 0.02 |
|  | Organic acids | O-Phosphoethanolamine | 1.80 | 0.04 |
|  | Purines and derivatives | Uric acid | 1.23 | 0.02 |
|  | Pyrimidines and pyrimidine derivatives | Uracil | 1.12 | 0.01 |
| P-BPH versus Healthy | 1,3,5-triazinanes | Hexamethylenetetramine | 0.95 | 0.01 |
|  | Amines | Linoleoyl ethanolamide | 1.30 | 0.01 |
|  |  | Dimethylethanolamine | 1.18 | 0.04 |
|  | Amines and derivatives | LysoPE(0:0/16:0) | 2.35 | 0.00 |
|  |  | LysoPE(0:0/16:1(9Z)) | 2.03 | 0.01 |
|  |  | LysoPE(18:1(9Z)/0:0) | 1.51 | 0.00 |
|  |  | LysoPE(0:0/18:2(9Z,12Z)) | 1.40 | 0.02 |
|  | Amino acids | L-Phenylalanine | 1.31 | 0.03 |
|  |  | L-Threonine | 1.17 | 0.04 |
|  |  | L-Tyrosine | 1.11 | 0.00 |
|  | Amino acids, peptides, and analogues | Threonylphenylalanine | 3.68 | 0.02 |
|  |  | Leucylleucine | 3.45 | 0.02 |
|  |  | Histidyltyrosine | 2.41 | 0.04 |
|  |  | Phenylalanylphenylalanine | 2.39 | 0.04 |
|  |  | gamma-Glutamylleucine | 1.81 | 0.00 |
|  |  | epsilon-(gamma-Glutamyl)lysine | 1.65 | 0.01 |
|  |  | Glycyl-L-leucine | 1.63 | 0.04 |
|  |  | D-Alanyl-D-alanine | 1.47 | 0.03 |
|  |  | Valylglycine | 1.38 | 0.01 |
|  |  | N-Acetyl-L-aspartic acid | 1.30 | 0.01 |
|  |  | gamma-Glutamylvaline | 1.21 | 0.03 |
|  |  | Glycylproline | 1.21 | 0.01 |
|  |  | N6-Acetyl-L-lysine | 1.12 | 0.02 |
|  |  | D-(+)-Proline | 1.07 | 0.01 |
|  |  | gamma-Glutamylisoleucine | 0.91 | 0.04 |
|  |  | 2-Aminoadipic acid | 0.88 | 0.02 |
|  |  | Symmetric dimethylarginine | 0.82 | 0.04 |
|  |  | Betaine | -1.10 | 0.01 |
|  | Antibiotics | Oleandomycin | 2.60 | 0.01 |
|  | Bile acids, alcohols and derivatives | 3beta-Hydroxy-5-cholestenoic acid | 0.96 | 0.03 |
|  | Carbohydrates | N-Acetylneuraminic acid | 2.26 | 0.04 |
|  |  | N-Acetyl-b-glucosaminylamine | 1.59 | 0.02 |
|  |  | N-Acetyl-D-glucosamine | 0.87 | 0.02 |
|  | Carbonyl compounds | 3,4-Dihydroxybenzaldehyde | -0.72 | 0.02 |
|  | Fatty acyls[FA] | Adrenic acid | 5.47 | 0.01 |
|  |  | Eicosapentaenoic acid | 1.59 | 0.00 |
|  |  | 8Z,11Z,14Z-Eicosatrienoic acid | 1.53 | 0.00 |
|  |  | Arachidonic acid | 1.50 | 0.02 |
|  |  | 8(S)-Hydroxy-(5Z,9E,11Z,14Z)-eicosatetraenoic acid | 1.46 | 0.01 |
|  |  | Oleic acid | 1.35 | 0.00 |
|  |  | Linoleic acid | 1.29 | 0.00 |
|  |  | Butyrylcarnitine | 1.13 | 0.04 |
|  |  | Cerebronic acid | 1.12 | 0.01 |
|  |  | Nervonic acid | 1.10 | 0.02 |
|  |  | 9-HOTrE | 0.96 | 0.01 |
|  |  | 2(R)-hydroxydocosanoic acid | 0.94 | 0.02 |
|  |  | 16(R)-HETE | 0.94 | 0.05 |
|  |  | cis-7-Hexadecenoic acid | 0.91 | 0.02 |
|  |  | 2-Hydroxyeicosanoic acid | 0.90 | 0.02 |
|  |  | Methylglutaric acid | -0.79 | 0.01 |
|  |  | Adipic acid | -0.93 | 0.04 |
|  |  | 3-Hydroxydecanoic acid | -0.98 | 0.01 |
|  |  | Valeric acid | -1.03 | 0.03 |
|  |  | (R)-3-Hydroxy myristic acid | -1.35 | 0.01 |
|  |  | 3-Carboxy-4-methyl-5-propyl-2-furanpropionic acid | -1.48 | 0.00 |
|  | Glycerolipids[GL] | MG(0:0/16:1(9Z)/0:0) | 1.20 | 0.00 |
|  |  | MG(18:1(9Z)/0:0/0:0) | 1.15 | 0.00 |
|  |  | 2-Arachidonoyl glycerol | 0.88 | 0.04 |
|  | Glycerophospholipids[GP] | LysoPC(P-18:0/0:0) | 1.95 | 0.03 |
|  | Nucleic acids and analogues | 5'-Methylthioadenosine | -0.69 | 0.03 |
|  | Organic acids | N1,N8-Diacetylspermidine | 1.30 | 0.02 |
|  |  | Ethyl 3-hydroxybutyrate | -0.59 | 0.04 |
|  |  | 12-Hydroxydodecanoic acid | -1.14 | 0.04 |
|  | Phenylpropanoic acids | Hydroxyphenyllactic acid | 0.61 | 0.01 |
|  | Phenylpropanoids | trans-Anethole | -1.01 | 0.00 |
|  | Purines and derivatives | Oxypurinol | 1.42 | 0.02 |
|  |  | Uric acid | 1.15 | 0.03 |
|  |  | Etamiphylline | -0.65 | 0.03 |
|  |  | Olomoucine | -0.85 | 0.02 |
|  |  | Nicotinic acid | 1.72 | 0.01 |
|  | Pyrimidines and pyrimidine derivatives | Orotic acid | 1.19 | 0.04 |
|  | Terpenoids | Betulin | 0.64 | 0.01 |
|  |  | Genipin | -1.24 | 0.02 |
